# Effects of Gentiopicroside on activation of NLRP3 inflammasome in acute gouty arthritis mice induced by MSU

**DOI:** 10.1007/s11418-021-01571-5

**Published:** 2021-09-29

**Authors:** Menglin He, Cheng Hu, Meijuan Chen, Qian Gao, Liqiu Li, Weiqian Tian

**Affiliations:** 1 Department of Anesthesiology, Affiliated Hospital of Nanjing University of Chinese Medicine, Jiangsu Province Hospital of Chinese Medicine, No. 155 Hanzhong Road, Qinhuai District, Nanjing, 210029 Jiangsu China; 2 School of Medicine and Holistic Integrative Medicine, Nanjing University of Chinese Medicine, Nanjing, 210023 Jiangsu China

**Keywords:** Gentiopicroside, Inflammation, Acute gouty arthritis, NLRP3 inflammasome

## Abstract

Acute gouty arthritis is a self-limiting inflammatory disease resulting from the deposition of monosodium urate (MSU) crystals. It has been shown that Gentiopicroside (GPS) possesses anti-inflammatory and analgesic functions. The aim of this study was to parse out whether GPS has an effect on acute gouty arthritis. We established an acute gouty arthritis model by the injection of MSU into the paw, and found that GPS relieves MSU-induced mechanical, thermal hyperalgesia, and paw swelling. Furthermore, GPS down-regulated the release of pro-inflammatory cytokines in paw tissues, including IL-1β, IL-6, IL-18, and TNF-α. The results of H&E staining and MPO activity measurement showed that GPS inhibits neutrophil infiltration. And the over-expressions of NOD-like receptor protein 3 (NLRP3), apoptosis-associated speck-like protein containing a caspase recruitment domain (ASC), and Caspase-1 induced by MSU were inhibited by treatment with GPS. These results revealed that GPS can treat acute gouty arthritis based on anti-inflammatory and analgesic properties in vivo, which might be ascribed to the inhibition on NLRP3 inflammasome. Furthermore, we performed in vitro study to confirm the results of in vivo study. Consistently, the results proved that GPS could inhibit the activation of NLRP3 inflammasome in RAW264.7 macrophages stimulated by LPS-MSU. In conclusion, this study provides an experimental basis for the application of GPS and expands the potential value of GPS in the therapy of acute gouty arthritis.

## Introduction

Gout is a common and reversible auto-inflammatory disease, which is characterized by the disorder of the innate immune and causes systemic inflammatory response [1]. It has been reported that gout is the most conventional type of inflammatory arthritis globally [2]. Uric acid is a physiological substance in our body, which has an antioxidant ability in low concentration [3]. However, high concentration of urate is identified as the key factor required for MSU crystallization [4]. And some patients with the deposition of MSU crystals manifest gout flare with symptoms of pain, swollen, and hot joint [5]. Additionally, the joints affected by gout are characterized by obvious neutrophil infiltration [6]. Multiple medications have been applied to treat acute gouty arthritis, including NASIDs, glucocorticoid and colchicine. But these treatments have inevitable limitations in clinic [7].

MSU crystals are first phagocytized by macrophages, and then promote the assembly and activation of NLRP3 inflammasome [8]. The activation of NLRP3 inflammasome needs NLRP3 to recruit the adaptor ASC, which then recruits caspase-1, resulting in the formation of inflammasome complex ultimately [9]. As an effector, caspase-1 cleaves the pro-IL-1β and pro-IL-18 into IL-1β and IL-18, respectively [10]. Furthermore, IL-1β elevates the expression of genes, which are related to fever, pain threshold, and promote immune cells to infiltrate infected or damaged tissues [11]. And IL-18 is a co-stimulatory cytokine that regulates adaptive immunity [12]. Research [13] demonstrated that multiple pro-inflammatory cytokines, such as IL-6 and TNF-α, are related to gouty arthritis. Therefore, the inhibition on NLRP3 inflammasome and the release of pro-inflammatory cytokines are crucial targets in the treatment of acute gouty arthritis.

GPS, as an iridoid glucoside separated from the root of *Gentiana Macrophylla Pall*, has been widely used to cure several diseases based on anti-inflammatory, hepato-protective, and anti-cancer properties [14–16]. GPS has been reported to inhibit the inflammation response in rat articular chondrocytes induced by IL-1β [17] and colitis in mice induced by dextran sodium sulfate [18]. Research [19] also demonstrated that GPS could inhibit the secretion of inflammatory factors. Furthermore, a recent study has studied the protective effect of GPS on ethanol-induced gastric mucosal injury, and the mechanism of effect was related to anti-inflammatory [20].

The effects of GPS have been illustrated in the above findings explicitly. However, the relation between GPS and acute gouty arthritis remains unknown. Therefore, the present study aimed to shed the light on the mechanism of GPS in treating acute gouty arthritis. We evaluated the effects of GPS on pain, paw swelling, and neutrophil infiltration in MSU-induced acute gouty arthritis mice. The inhibition of GPS on the release of pro-inflammatory cytokines was also explored. Furthermore, the activation of NLRP3 inflammasome was analyzed both in vivo and vitro studies.

## Materials and methods

### Experimental animals

Specific-pathogen-free male C57BL/6 mice (18–22 g, 6–8 weeks of age) were purchased from Qinglong Mountain Animal Breeding Farm, Nanjing, China. The mice were housed in plastic cages and kept on a 12/12 h light/dark cycle and were given access to food and water with no restrictions in a room with an ambient temperature of 22 °C ± 2 °C during the experiment. All animal procedures were carried out in strict accordance with the guideline of the National of Health Guide for the Care and Use of Laboratory Animals.

### Cell culture and treatment

RAW264.7 macrophage cell line was purchased from Shanghai Fuheng Biology (Shanghai, China) and cultured in DMEM (Gibco, USA) supplemented with 10% FBS (Hycolon, USA) at 37 °C with 5% CO_2_ and 95% air. After preconditioning with or without LPS at the concentration of 200 μg/ml for 4 h, the cells were treated with GPS (1250, 2500, and 5000 μM) followed by exposure to 500 ug/ml MSU for 4 h. LPS, GPS, and MSU were dissolved in PBS, and MSU solution was processed with Ultrasonic Liquid Processors before application (QSONIC, USA).

### Cell viability assay

The Cell Counting Kit-8 was applied to assess the cytotoxicity of GPS. Primarily, 5 × 10^3^ RAW264.7 cells were plated per well of a 96-well plate followed by 24 h incubation. Then cells were treated with a range of GPS concentrations for another 24 h in 100 μL medium. The medium was removed, CCK-8 reagent and fresh medium were added to per well at 1:10 ratio in a 2 h incubation period at 37 °C. The absorbance in 450 nm was measured spectrophotometrically using a microplate reader (BioTek, Santa Barbara, CA, USA).

### Reagents

GPS (purity > 97%, #20831-76-9) was purchased from Yuanye Bio-Technology (Shanghai, China). GPS was dissolved in PBS as a stock solution at 1 g/ml and stored at 4 °C before use. Lipopolysaccharide (LPS) (L2630) was purchased from Sigma–Aldrich (USA). Antibodies against NLRP3 (AG-20B-0014-C100) and Caspase-1 (AG-20B-0042-C100) were purchased from Adipogen Life Sciences (San Diego, USA). Antibodies against ASC (#67824) and β-actin (#3700) were obtained from Cell Signaling Technology (Cell Signaling, Danvers, Technology, MA, USA). Horseradish peroxidase (HRP)-conjugated goat anti-mouse (SA00001-1), and HRP-conjugated goat anti-rabbit (SA00001-2) antibodies were purchased from proteintech (Boston, MA, USA). The BCA Protein Assay Kit was obtained from Beyotime (Beyotime Institute of Biotechnology, Nanjing, China).

### Preparation of MSU crystals

**Fig. 1:**
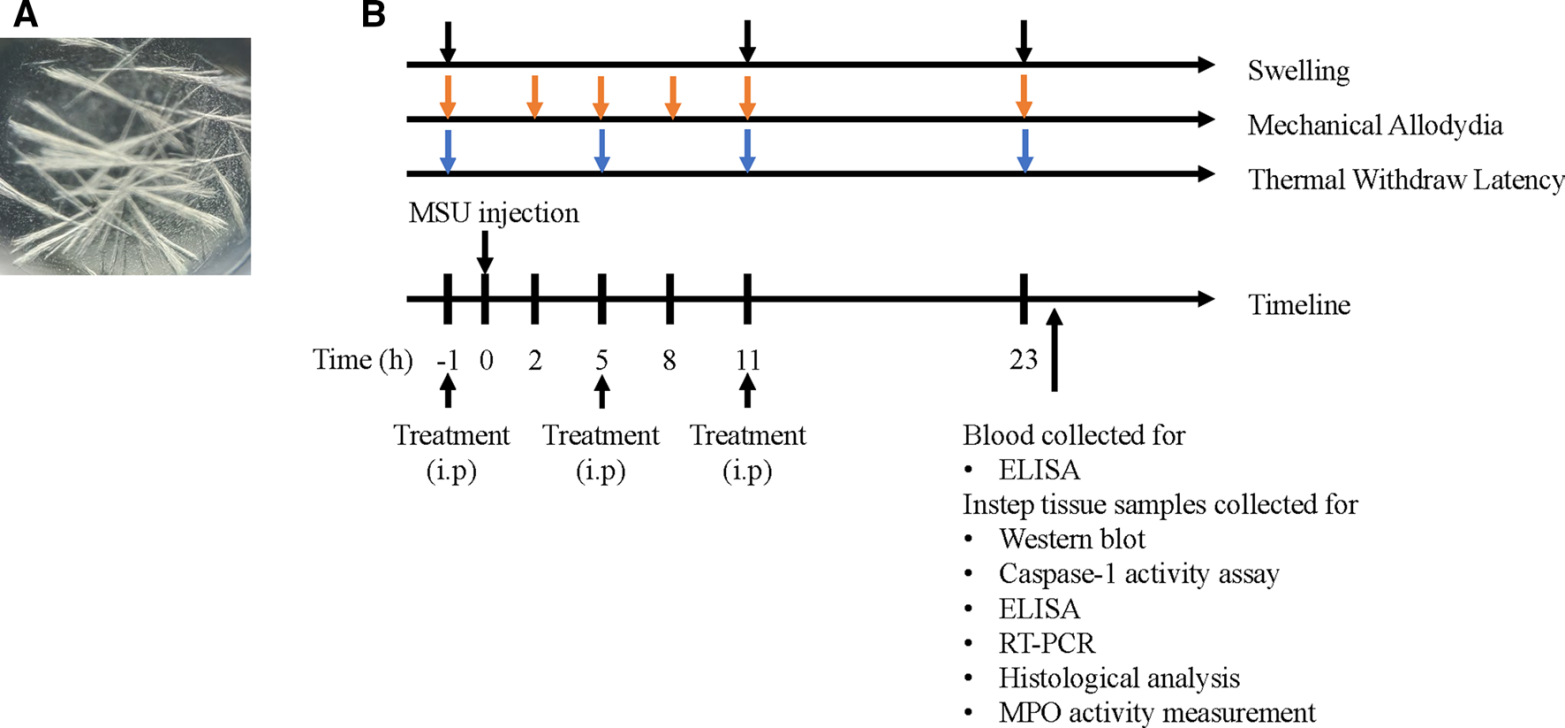
The structure of MSU crystals and experimental protocol. **a** The structure of MSU crystals in polarizing microscope. **b** The experimental protocol: MSU crystals (0.5 mg MSU in 20 μL PBS) or PBS (20 μL) was applied into the instep of right paw to establish the acute gouty arthritis model or control group. Swelling was measured at 1 h before MSU injection and 11, 23 h after model establishment. Mechanical allodynia was detected 1 h before MSU injection and 2, 5, 8, 11, 23 h after MSU injection. The measurement of thermal withdraw latency was conducted at 1 h before MSU injection and 5, 11, 23 h after model establishment. Mice were killed to collect blood and instep tissue for Western blot, Caspase-1 activity assay, ELISA, RT-PCR, Histological analysis, and MPO activity measurement

Uric acid (800 mg) was added into boiling water (155 mL) containing 1 N NaOH (5 mL). And the PH of solution was altered to 7.2 with hydrochioric acid (HCl). MSU crystals were prepared after autoclaved sterilization. The whole procedures followed the previous study [21]. The structure of MSU crystals was visualized (Fig. 1a).

### Establishment and treatment of acute gouty arthritis model

48 male C57BL/6 mice were randomly divided into six groups. GPS (50, 100, 200 mg/kg), colchicine (1 mg/kg) or vehicle (0.9% normal saline) were applied intraperitoneal. 1 h after pretreatment with colchicine and GPS, all groups were injected with MSU crystals (0.5 mg MSU crystals dissolved in 0.02 ml PBS) into the instep of the left hind leg under isoflurane anesthesia, while the control group was injected with the same volume of PBS. Treatment with GPS was conducted repeatedly at 5 and 11 h after MSU injection.

### Evaluation of swelling

To assay the inflammation response of the instep, the circumference of the instep was measured by a graduated rope. Overall, a total of three measurements was conducted in the entire experiment, and the measurement result before MSU stimulation was used as the standard circumference. The next two measurements were performed at 11 and 23 h after the injection of MSU. Swelling index = (circumference of the instep after MSU stimulation − standard circumference)/standard circumference.

### Evaluation of mechanical allodynia

Mice were individually placed in the transparent glass boxes for 15–20 min to adapt to the environment before evaluation. In the bottom of the boxes, Von Frey calibrated filaments (UGO Basile, Italy) were used to detect the plantar surface of the left hind leg through the wire mesh, bending the filaments slightly for 3–5 s with enough force. Increasing filament stiffness if mice had no reaction or decreasing filament stiffness if mice appeared abrupt withdraw of the paw, licking or vigorously shaking. The intensity of the pressure was recorded. The up–down testing paradigm and nonparametric Dixon test were performed to calculate the 50% paw withdrawal threshold (PWT) [22, 23]. The baseline of PWT was measured 1 h before the injection of MSU, and mechanical allodynia were frequently assessed at 2, 5, 8, 11, and 23 h after the injection of MSU to explore the time effect of GPS and MSU.

### Evaluation of thermal withdraw latency

Plantar test (UGO Basile, Varese, Italy) was applied to test thermal sensitivity as described previously [24]. Mice were placed in glass boxes individually for 15 min to acclimate the environment before the test. In brief, the hind paw was exposed to the radiant emission by the light source. The time of paw licking or withdrawal, which were considered as a response to avoid thermal pain, was recorded. The cut-off time was set as 30 s to avoid burns on the plantar skin of mice. We conducted three measurements with 10-min interval and the average of the three measurements was regarded as the final result. We performed tests at 1 h before and 5, 11, 23 h after the injection of MSU, respectively.

### Blood processing

After mice were anesthetized by 1% sodium pentobarbital, blood samples were collected and kept at room temperature for about 1 h to ensure the separation of serum. Then the serum was collected through centrifugation at 5000 rpm for 15 min at 37 °C. Serum samples were used for the cytokines assays for ELISA and stored at − 80 °C before other assays.

### Histological analysis

The instep tissue samples were collected after last evaluation, and fixed in the 4% paraformaldehyde solution. Then, the samples were decalcified for 20 days with EDTA and embedded with paraffin. Subsequently, the paraffin sections were stained with hematoxylin and eosin for conventional morphological assessment using a light microscope on 40 × objective.

### Myeloperoxidase (MPO) activity measurement

The MPO Detection Kit (Nanjing JianCheng Bio Ins, China) was used to evaluate the quantification of neutrophil infiltration in the paw tissue as described previously [25]. Briefly, mice were anaesthetized, and the paw tissue was homogenized and centrifuged at 12,000 r/min at 4 °C for 20 min. 10 μL of the supernatant was transferred into pbs (pH 6.0) containing 0.17 mg/ml 3,3′-dimethoxybenzidine and 0.0005% H_2_O_2_. MPO can catalyze the redox reaction of hydrogen peroxide with 3,3′,5,5′-tetramethylbenzidine, which then produce a yellow compound. MPO activity was calculated by measuring the value of product in 460 nm with spectrophotometer and expressed as units per gram of total protein (u/g).

### Caspase-1 activity assay

The Caspase-1 Activity Kit (Beyotime Institute of Biotechnology, Nanjing, China) was used to assay the activity of Caspase-1 according to the manufacturer’s protocol. This assay is based on the function of Caspase-1 that can convert acetyl-Tyr-Val-Ala-Asp p-nitroanilide (Ac-YVAD-pNA) into the yellow formazan product p-nitroanilide (p-NA). Tissue lysates were centrifuged at 14,000*g* for 10 min, and the protein concentrations were determined by the Bradford protein assay. A total of 50 μg of protein was incubated in a 96-well microtiter plate with 20 ng of Ac-DEVD-pNA overnight at 37 °C. The absorbance value of pNA at 405 nm was measured using a 96-well plate reader. The increase of pNA value indicates the activation of caspase-1.

### Western blot

Paw samples and cells were lysed in a RIPA buffer containing 1 mM phenylmethylsulfonyl fluoride (PMSF) and further centrifugated at 14,000×*g* for 15 min at 4 °C. The supernatant was obtained and its total protein concentration was measured by a BCA protein assay kit. Protein was loaded and separated by 8–12% sodium dodecyl sulfate–polyacrylamide gel electrophoresis (SDS–PAGE), then polyvinylidene fluoride (PVDF) membranes were applied to transfer protein. The membranes were blocked with 5% (w/v) non-fat milk in TBST buffer on the shaker for 2 h at room temperature followed by incubation with corresponding primary antibodies (1:1000 dilution) overnight at 4 °C, and incubated with HRP-conjugated secondary antibodies (1:4000 dilution). Finally, antigen–antibody complexes were detected by ECL chemiluminescent substrate. The levels of protein expression are normalized to the density of β-actin.

### Real-time quantitative PCR

The paw tissue samples at 23 h after the establishment of acute gouty arthritis and cells were collected and extracted in 1 mL Trizol reagent (Invitrogen, USA) for total RNA followed by centrifugation at 12,000 rpm for 10 min at 4 °C. The purity and concentration of the samples were measured using a spectrophotometer, and the standard of wavelength absorption ratio (260/280 nm) was set between 1.8 and 2.0. cDNA was obtained using HiScript III RT SuperMix (Vazyme Biotech Co., Ltd. China) according to the manufacturer’s instructions. Gene expression was determined by Stratagene MX3000P using ChamQ Universal SYBR qPCR Master Mix (Vazyme Biotech Co., Ltd. China). Relative mRNA expression was calculated using 2^−ΔΔCt^ method and GAPDH was used as reference gene. The qPCR primers are listed in Table 1.

**Table 1:** Sequences of the primers used for qPCR

Genes	Sequences of primers
NLRP3	F: 5′ GCCGTCTACGTCTTCTTCCTTTCC 3′ R: 5′ CATCCGCAGCCAGTGAACAGAG 3′
ASC	F: 5′ ACAATGACTGTGCTTAGAGACA 3′ R: 5′ CACAGCTCCAGACTCTTCTTTA 3′
Caspase-1	F: 5′ AGAGGATTTCTTAACGGATGCA 3′ R: 5′ TCACAAGACCAGGCATATTCTT 3′
GAPDH	F: 5′ CTCTCTGCTCCTGTTCGACAG 3′ R: 5′ GTGTAATCATATTGGAACATGTAG 3′

### Enzyme-linked immunosorbent assay

Serum and paw tissue were collected to detect the quantity of TNF-α (ZC-39024), IL-1β (ZC-37974), IL-6 (ZC-37988), and IL-18 (ZC-37973) using corresponding ELISA kits according to the manufacturer’s instructions (ZCIBIO Technology Co. Ltd).

### Statistical analyses

Data were analyzed with GraphPad Prism 7 (GraphPad Software, La Jolla, CA, USA) and presented as the mean ± SD. Statistical significance was determined by Student’s *t* test or one-way analysis of variance (ANOVA) followed by Bonferroni test. *P* < 0.05 was considered statistically significant.

## Results

### The therapeutic effects of GPS on MSU-induced pain and swelling

**Fig. 2:**
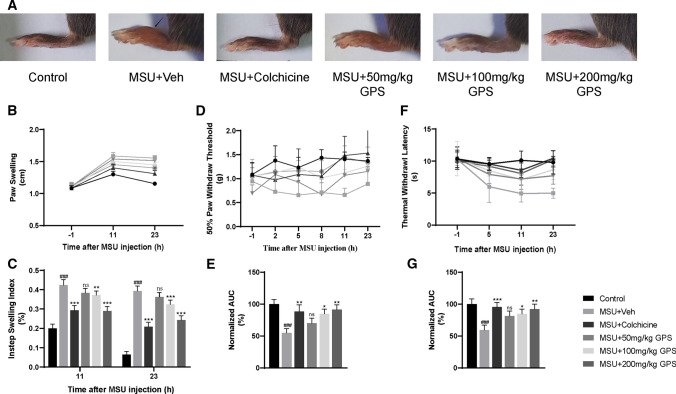
Effects of GPS on swelling and pain in acute gouty arthritis induced by MSU. **a** Representative photographs of ankles 24 h after MSU injection. Black arrow indicates the injected place. **b** Time courses of the effects of colchicine and different doses of GPS on paw swelling. *n* = 6 mice per group. **c** Comparison of the instep swelling indexes at different points after MSU injection in each group. **d**, **f** Time course of the effects of colchicine and different doses of GPS on mechanical and thermal allodynia. **e**, **g** Normalized AUC of **d**, **f**. Values are expressed as means ± SD. ^###^*p* < 0.001 vs. the control group; *p < 0.05 vs. the MSU + Veh group; **p < 0.01 vs. the MSU + Veh group. ***p < 0.001 vs. the MSU + Veh group. ns, not significantly different from MSU + Veh group; one-way or two-way ANOVA followed by Tukey’s post hoc test

Pain and swelling are the remarkable symptoms in patients with acute gouty arthritis in clinic [26]. Therefore, we established acute gouty arthritis model induced by MSU to analyze the effects of GPS on these signs. Evaluations were conducted at different times (Fig. 1b). We took paw swelling as an indicator of the effects of GPS. Figure 2a, b showed that GPS and colchicine relieve paw swelling induced by MSU. Compared with MSU + Veh group, 100 mg/kg and 200 mg/kg GPS groups showed significant inhibition on instep swelling index (Fig. 2c). Next, we evaluated the effects of GPS on MSU-induced pain. The results showed that the severity of pain induced by MSU achieved its peak at 8 h after stimulus. And 100 mg/kg, 200 mg/kg GPS had analgesic effect on mechanical hyperalgesia, while the 50 mg/kg GPS did not show (Fig. 2d, e). Furthermore, MSU-induced a severe thermal hyperplasia, which was inhibited by the treatment of GPS with 100 mg/kg and 200 mg/kg (Fig. 2f, g).

### GPS inhibited NLRP3 inflammasome activation in vivo and vitro studies

**Fig. 3:**
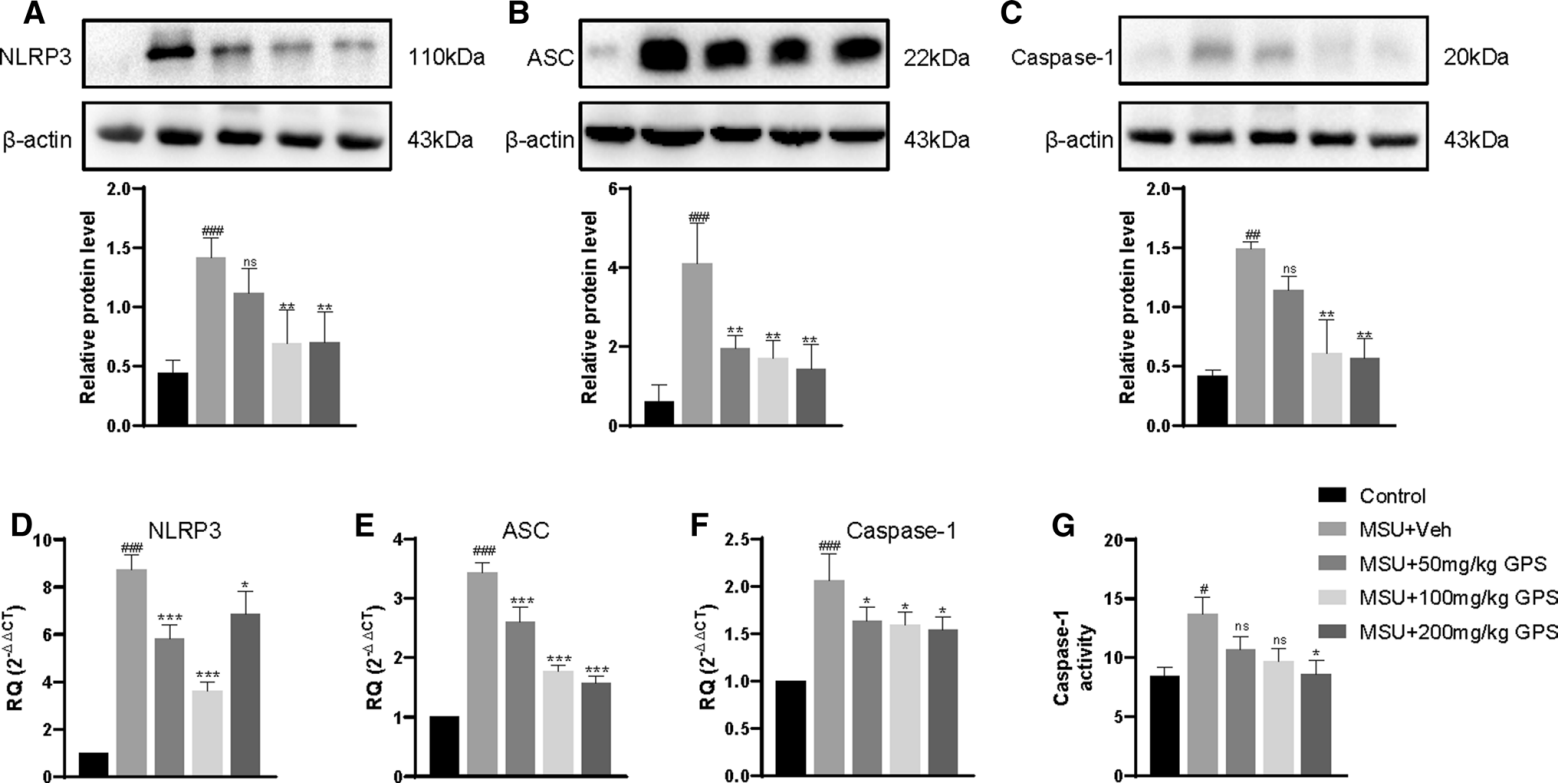
GPS inhibited the activation of NLRP3 inflammasome in paw tissue of acute gouty arthritis. NLRP3 (**a**), ASC (**b**) and Caspase-1 (**c**) protein expressions were evaluated 24 h after MSU injection. n = 6 mice per group. Measurements of NLRP3/β-actin, ASC/β-actin, and Caspase-1/β-actin were quantified by Image J software. **d**–**f** The mRNA levels of NLRP3, ASC, and Caspase-1 in paw tissue were determined by qPCR. **g** The activity of Caspase-1 in paw tissues of MSU-induced acute gouty arthritis mice was determined by the Caspase-1 Activity Kit. Values are expressed as means ± SD. ^#^*p* < 0.05 vs. the control group; ^##^*p* < 0.01 vs. the control group; ^###^*p* < 0.001 vs. the control group; *p < 0.05 vs. the MSU + Veh group; **p < 0.01 vs. the MSU + Veh group. ***p < 0.001 vs. the MSU + Veh group. ns, not significantly different from MSU + Veh group

To further investigate whether the mechanism of GPS to treat acute gouty arthritis is related to NLRP3 inflammasome, we first performed western blot to analyze the activation of NLRP3 inflammasome in vivo study. After MSU stimulation, the expressions of NLRP3, ASC, and Caspase-1 were upregulated. But these effects were significantly inhibited by GPS (Fig. 3a–c). Next, we examined the effects of GPS on NLRP3 inflammasome activation by qPCR. Consistent with aforementioned results, the results of qPCR demonstrated that GPS significantly attenuated the over-expressions of NLRP3, ASC, and Caspase-1 mRNA in paw tissues induced by MSU (Fig. 3d–f). We further used the Caspase-1 Activity Kit to verify our results. Coherently, the results showed that GPS can downregulate the activity of caspase-1 (Fig. 3g).

**Fig. 4:**
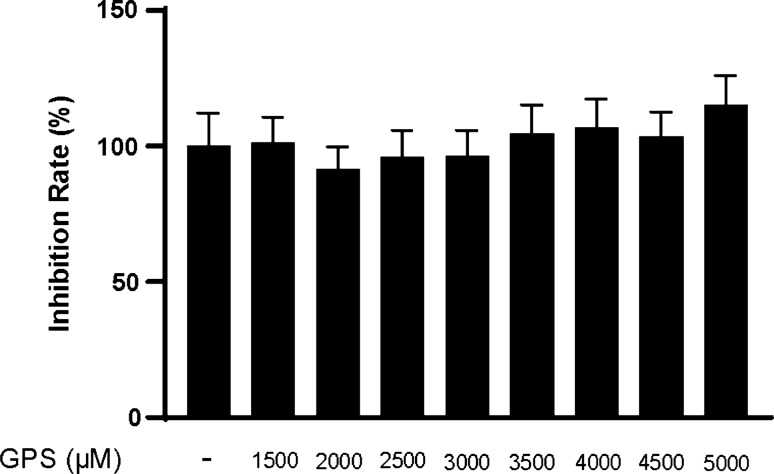
Effects of GPS-induced cytotoxicity in RAW264.7 macrophages. All data were presented as mean ± SD. There was no difference between groups

**Fig. 5:**
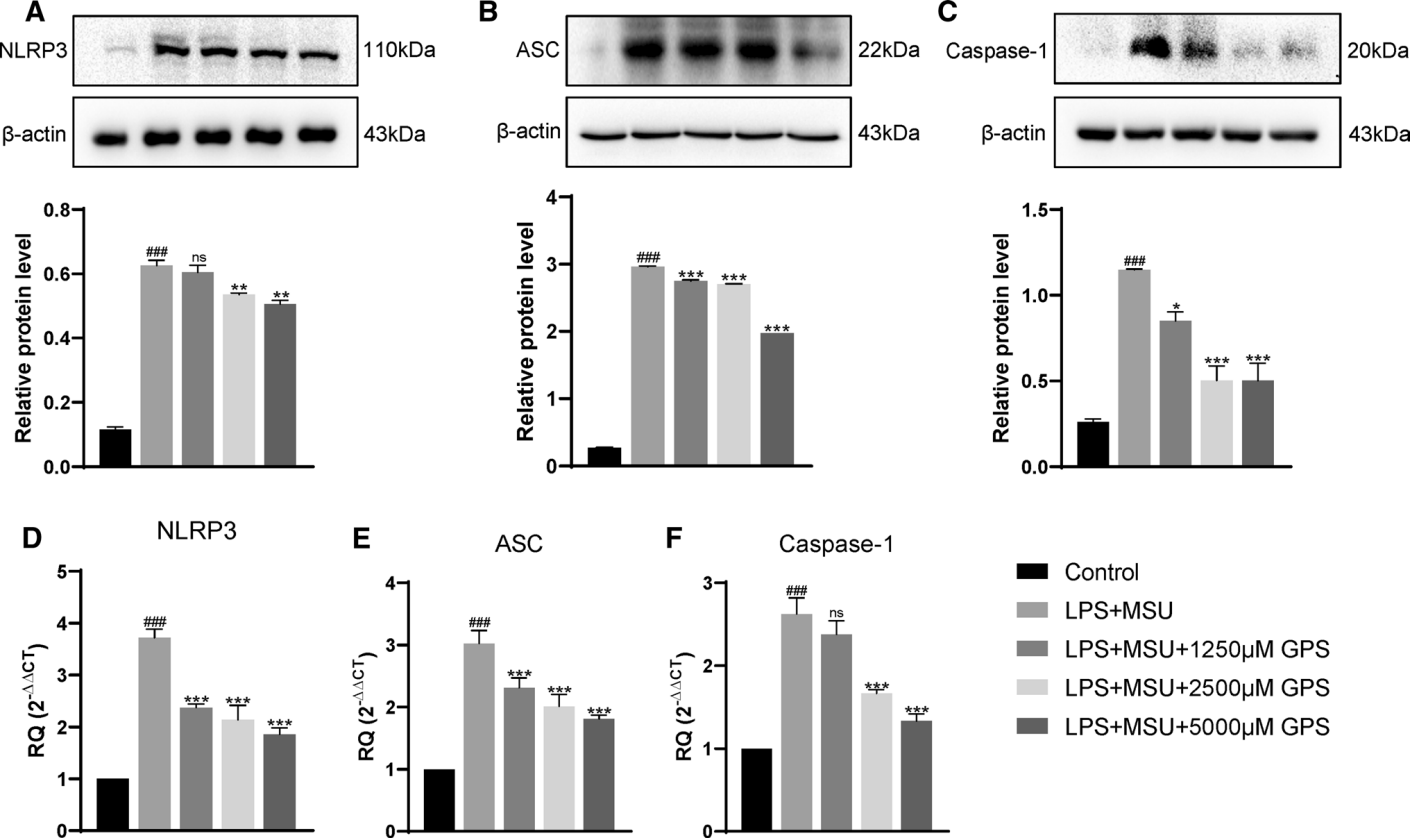
GPS inhibited the activation of NLRP3 inflammasome in RAW264.7 macrophages induced by LPS-MSU. NLRP3 (**a**), ASC (**b**) and Caspase-1 (**c**) protein expressions were evaluated. Measurements of NLRP3/β-actin, ASC/β-actin and Caspase-1/β-actin were quantified by Image J software. (D,E,F) The mRNA levels of NLRP3, ASC, Caspase-1 in RAW264.7 macrophages were determined by qPCR. Values are expressed as means ± SD. ^###^*p* < 0.001 vs. the control group; *p < 0.05 vs. the MSU + Veh group; **p < 0.01 vs. the MSU + Veh group. ***p < 0.001 vs. the MSU + Veh group. ns, not significantly different from MSU + Veh group

Furthermore, we conducted in vitro study to confirm the inhibition of GPS on the activation of NLRP3 inflammasome. To choose the optimal concentrations of GPS in this study, the CCK-8 assay was performed. The results demonstrated that GPS showed no significant toxicity to RAW264.7 macrophages at concentration below 5000 μM after 24 h (Fig. 4). Similarly, we assessed the level of crucial components of the NLRP3 inflammasome in RAW264.7 macrophages. The results showed that the expressions of NLRP3, ASC, and Caspase-1 were inhibited by GPS in dose-dependent (Fig. 5a–c). The mRNA levels of NLRP3, ASC, and Caspase-1 induced by LPS and MSU were also inhibited by GPS (Fig. 5d–f).

### The inhibition of GPS on the release of pro-inflammatory cytokines and neutrophil infiltration

**Fig. 6:**
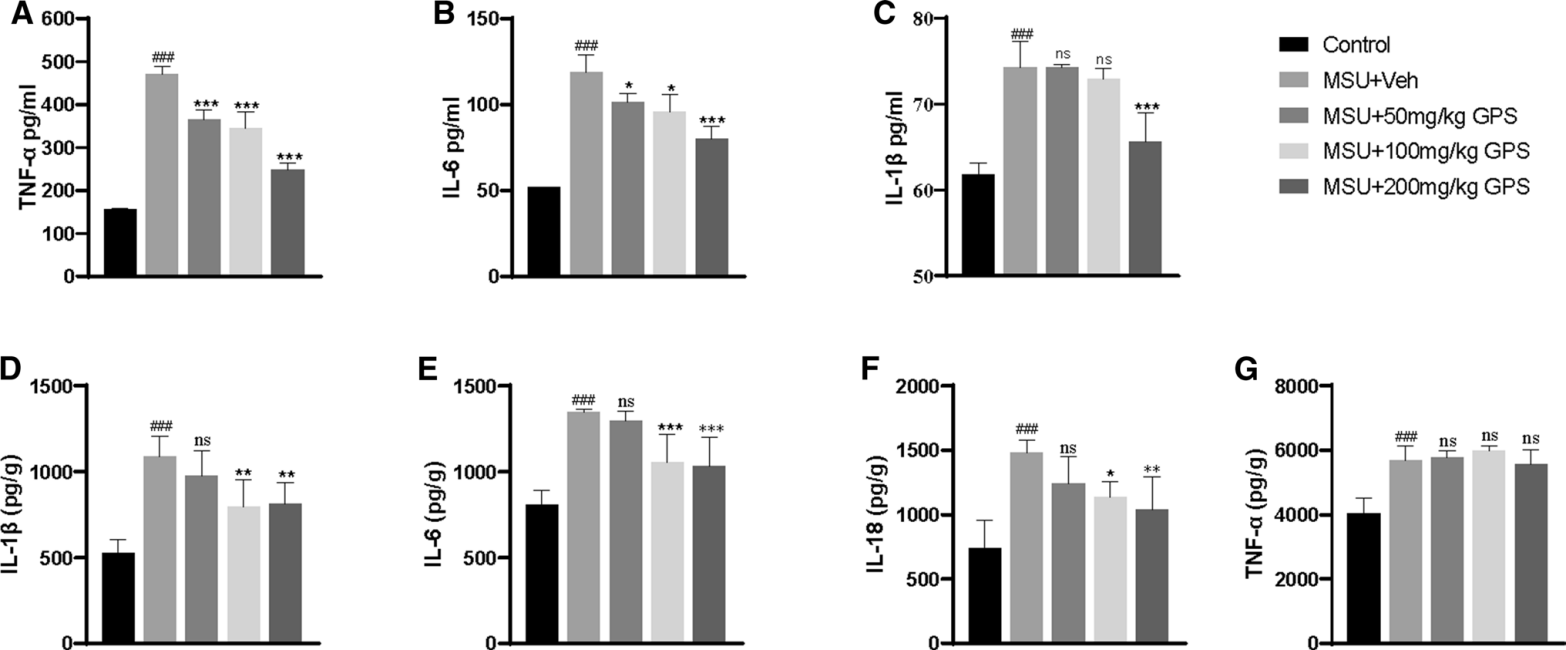
The inhibition of GPS on the release of pro-inflammatory cytokines, including IL-1β, IL-6, IL-18, and TNF-α in serum and paw tissue. **a**, **b** The TNF-α and IL-6 levels were markedly decreased by the treatment of GPS than the control group in serum. **c**–**e** The IL-1β, IL-6, and IL-18 levels were significantly reduced in MSU + 100 mg/kg and MSU + 200 mg/kg groups in paw tissue. Values are expressed as means ± SD. ^###^*p* < 0.001 vs. the control group; *p < 0.05 vs. the MSU + Veh group; **p < 0.01 vs. the MSU + Veh group. ***p < 0.001 vs. the MSU + Veh group. ns, not significantly different from MSU + Veh group

Considering the important effect of pro-inflammatory cytokines on the development of acute gouty arthritis, we explored whether GPS could inhibit the release of IL-1β, IL-6, IL-18, and TNF-α. We analyzed the expression levels of IL-1β, TNF-α, IL-6 in serum, and found that GPS decreased the expressions of TNF-α (Fig. 6a), IL-6 (Fig. 6b), and IL-1β (Fig. 6c). Then, the pro-inflammatory cytokines in the paw tissue were analyzed. The expression levels of IL-1β, IL-6, and IL-18 were inhibited by GPS except the group of 50 mg/kg GPS (Fig. 6d–f), and GPS had no effect on the expression level of TNF-α (Fig. 6g).

**Fig. 7:**
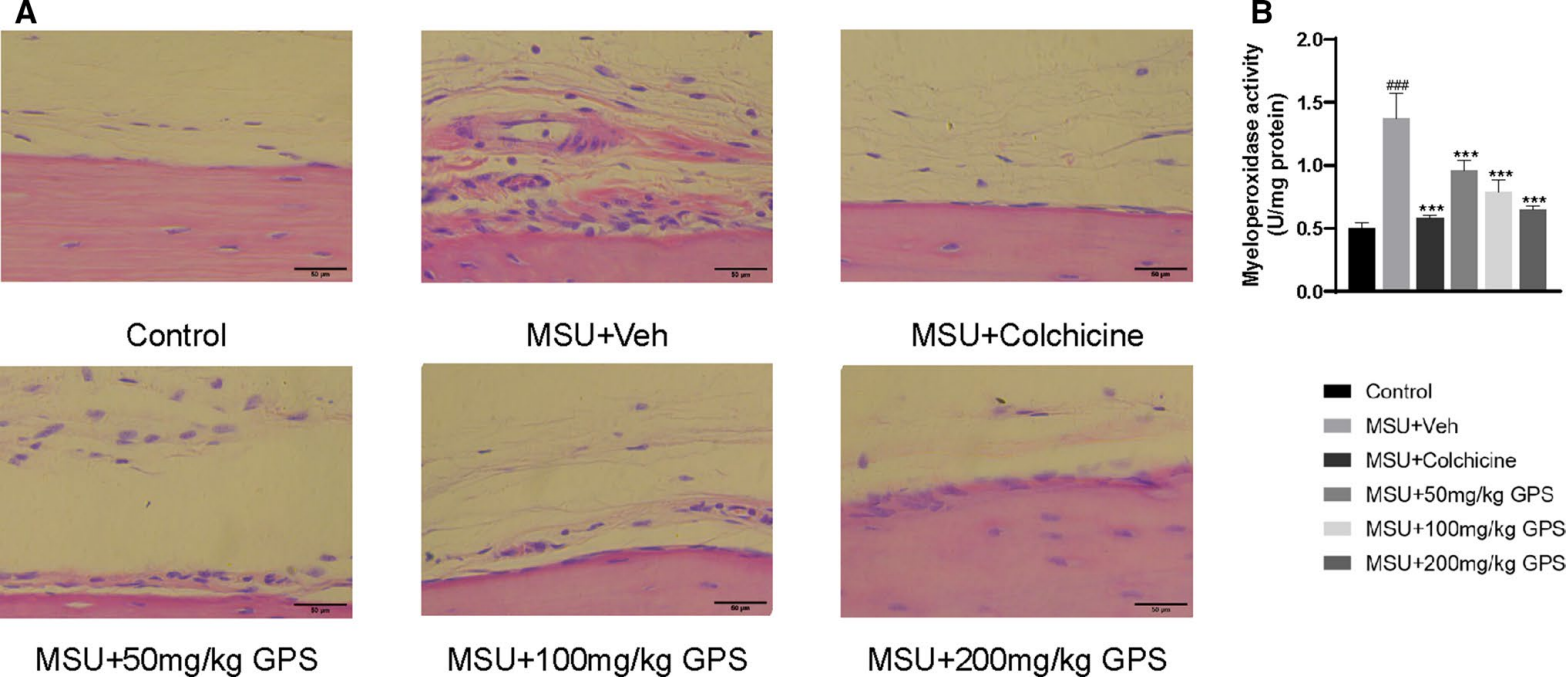
Effects of GPS on inflammatory cell infiltration in instep tissues in the model of acute gouty arthritis induced by MSU. **a** H&E staining (× 400) of inflammatory cells in instep tissues. **b** Myeloperoxidase (MPO) activity was determined in instep tissues. *n* = 6 mice per group. Values are expressed as means ± SD. ^###^*p* < 0.001 vs. the control group; ^***^p < 0.001 vs. the MSU + Veh group

We further examined the effects of GPS on inflammatory cell infiltration induced by MSU in paw tissue. MSU significantly induced the inflammatory cells infiltration in paw tissue. Furthermore, animals treated with GPS and colchicine progressed to less paw tissue lesion, as evidenced by significantly decreased infiltration of inflammatory cells in the instep of paw (Fig. 7a). Previous study [27] demonstrated that neutrophil depletion alleviates inflammation, indicating the significance of inhibition on the recruitment of neutrophil. Therefore, Myeloperoxidase (MPO) assay was performed to evaluate the effects of GPS on neutrophil infiltration in paw tissue, GPS and colchicine both significantly attenuated MSU-induced neutrophil infiltration in paw tissue (Fig. 7b).

## Discussion

In the present study, we parsed out the role of GPS in the model of acute gouty arthritis induced by MSU and in the RAW264.7 macrophages induced by LPS-MSU. The model aforementioned shares canonical characteristics of gout flares in humans. The injection of MSU-induced swelling, severe pain, pro-inflammatory cytokines release, the infiltration of neutrophil and the activation of NLRP3 inflammasome. Whereas, these effects were alleviated by the treatment with GPS. The activation of NLRP3 inflammasome in RAW264.7 cells induced by LPS-MSU was also inhibited by GPS. Above results demonstrated that GPS is a promising treatment for acute gouty arthritis.

GPS has been reported to have an obvious inhibition on swelling in arthritis induced by adjuvant [28]. Research [29] also demonstrated that GPS has an analgesic effect on mechanical, thermal, cold hyperalgesia in CCI model. And the effects of GPS on mechanical, thermal pain induced by MSU were indicated by our findings. Furthermore, we compared the effects of GPS with colchicine and found that they share similar effectiveness. Here, we demonstrated that GPS could effectively attenuate swelling and pain in acute gouty arthritis induced by MSU, which provides the first evidence displaying the therapeutic effects of GPS on acute gouty arthritis.

MSU crystals induce an inflammatory response, which is dependent on the activation of NLRP3 inflammasome [30]. Research [31] reported that NLRP3^−/−^, ASC^−/−^, and Caspase-1^−/−^mice alleviate MSU-induced ankle swelling as compared to WT mice. GPS has been shown to possess the inhibition on the expression of NLRP3, ASC, and Caspase-1 in adjuvant-induced arthritis [32]. In the present study, MSU-induced an obvious increase in NLRP3, ASC, Caspase-1, and IL-1β, and these growths were curbed by GPS treatment. Together, these results demonstrated that GPS is able to attenuate gouty arthritis via inhibiting NLRP3 inflammasome activation. However, further explorations of the mechanism of GPS on NLRP3 inflammasome are required.

Pro-inflammatory cytokines have been implicated in initiation and amplification of the gout flare, such as IL-1β, IL-6, and TNF-α [12]. The activation of NLRP3 inflammasome induces the release of IL-1β and IL-18. Afterwards, IL-1β promotes the expression of pro-inflammatory cytokines, including TNF-α and IL-6, leading to neutrophil infiltration [33]. In turn, neutrophils interact with MSU crystals and induce the release of IL-1, IL-6, TNF-α, and other cytokines, resulting in pain and erythema related to gout flare [34, 35]. Inhibition on TNF-α and IL-1β has been demonstrated to reduce neutrophil infiltration and pain in rheumatic disease [30, 36]. IL-18 is deemed to elevate cell adhesion molecules for leukocyte trafficking and induces chemokine production [37]. Therefore, it is effective to inhibit neutrophil infiltration induced by MSU and the release of pro-inflammatory cytokines in the management of acute gouty arthritis. Consistent with previous results [38], we verified that GPS can decrease the levels of TNF-α, IL-6 in serum and inhibit the release of IL-1β, IL-6, and IL-18 in the paw tissue. The neutrophil infiltration induced by MSU was also alleviated by GPS.

In conclusion, the present study indicated that GPS can attenuate MSU-induced pain, swelling, and neutrophil infiltration via inhibiting the release of pro-inflammatory cytokines. We further demonstrated that the effects of GPS are related to the inactivation of NLRP3 inflammasome in vivo and vitro study. To our knowledge, this is the first time to explore the mechanism of GPS in treating acute gouty arthritis. Therefore, GPS may be a novel effective strategy in treating acute gouty arthritis based on analgesic and anti-inflammatory properties.
